# Prenatal Cadmium Exposure and Maternal Sex Steroid Hormone Concentrations across Pregnancy

**DOI:** 10.3390/toxics11070589

**Published:** 2023-07-06

**Authors:** Zorimar Rivera-Núñez, Megan Hansel, Camila Capurro, Danielle Kozlosky, Christina Wang, Cathleen L. Doherty, Brian Buckley, Pamela Ohman-Strickland, Richard K. Miller, Thomas G. O’Connor, Lauren M. Aleksunes, Emily S. Barrett

**Affiliations:** 1 Department of Biostatistics and Epidemiology, Rutgers School of Public Health, Piscataway, NJ 08854, USA; mh1385@sph.rutgers.edu (M.H.); cc2091@sph.rutgers.edu (C.C.); ohmanpa@sph.rutgers.edu (P.O.-S.); esb104@eohsi.rutgers.edu (E.S.B.); 2 Environmental and Occupational Health Sciences Institute, Rutgers University, Piscataway, NJ 08854, USA; danielle.kozlosky@gmail.com (D.K.); cld133@eohsi.rutgers.edu (C.L.D.); bbuckley@eohsi.rutgers.edu (B.B.); aleksunes@eohsi.rutgers.edu (L.M.A.); 3 Clinical and Translational Science Institute, The Lundquist Institute at Harbor-UCLA Medical Center, Torrance, CA 90502, USA; wang@lundquist.org; 4 Department of Obstetrics and Gynecology, University of Rochester Medical Center, Rochester, NY 14620, USA; richardk_miller@urmc.rochester.edu (R.K.M.); tom_oconnor@urmc.rochester.edu (T.G.O.); 5 Department of Environmental Medicine, Pediatrics and Pathology, University of Rochester, New York, NY 14642, USA; 6 Departments of Psychiatry, Psychology, Neuroscience, University of Rochester, New York, NY 14620, USA; 7 Department of Pharmacology and Toxicology, Ernest Mario School of Pharmacy, Rutgers University, Piscataway, NJ 08854, USA

**Keywords:** cadmium, sex steroid hormones, pregnancy, androgens, testosterone

## Abstract

Cadmium exposure has been associated with adverse perinatal outcomes. One possible mechanism is endocrine disruption. Studies of non-pregnant adults suggest that cadmium impacts androgen production; here, we examined these associations during pregnancy. Participants in the Understanding Pregnancy Signals and Infant Development (UPSIDE) cohort provided biospecimens and questionnaire data in each trimester (n = 272). We quantified urinary cadmium, serum total testosterone (TT), estrone, estradiol, and estriol and serum free testosterone (fT). In adjusted longitudinal models, we examined sex steroid concentrations across pregnancy in relation to specific gravity-adjusted, ln-transformed cadmium concentrations. Additionally, we examined trimester-specific associations and stratified models by fetal sex. Results are presented as percent change (%∆) in hormone concentrations. In longitudinal models, higher cadmium concentrations were associated with lower fT across pregnancy (%∆ = −5.19, 95%CI: −8.33, −1.93), with no differences in other hormones observed. In trimester-specific models, higher cadmium concentrations were associated with lower TT in trimester 2 (%∆ = −15.26, 95%CI: −25.15, −4.06) and lower fT in trimester 3 (%∆ = −14.35, 95%CI: −19.75, −8.59). Associations with TT were stronger in pregnancies carrying female fetuses. Maternal cadmium exposure may be associated with reduced testosterone in pregnancy. Additional work is necessary to understand how alterations in gestational testosterone activity may impact pregnancy and child health.

## 1. Introduction

Cadmium is a toxic metal widely used in industrial processes and found in the environment due to industrial emissions, mining, and the burning of coal. It is commonly used in commercial products, such as batteries and petroleum-based plastics, and human exposure also occurs through use of tobacco products or consumption of contaminated food [1]. Exposure to cadmium has been linked to numerous adverse health outcomes, such as impaired airway function [2], cardiovascular disease [3], and kidney disease [4]. In the U.S. population, most people have detectable levels of cadmium in their bodies, including 83% of pregnant participants in the National Health and Nutrition Examination Survey (NHANES) [5]. This widespread exposure is important because, during pregnancy, cadmium exposure may have adverse impacts on fetal development. A 2021 meta-analysis of 18 studies estimated that for every 1 µg/L increase in maternal cadmium, there was a 42.11 g reduction in birthweight and a 0.11 cm reduction in head circumference at birth [6]. Prenatal cadmium exposure may also contribute to pregnancy complications including preterm labor [7], hypertensive disorders of pregnancy [8], and gestational diabetes [9], as well as adverse child outcomes such as developmental delays [10] and impaired immune function [11,12]. The mechanisms underlying associations between prenatal cadmium exposure and perinatal and child health outcomes remain poorly understood. Additionally, these mechanisms may differ considerably from other environmental exposures, given that in contrast to many other toxicants, relatively little cadmium crosses the placenta to directly impact the fetus in animals and human studies; instead, the placenta highly concentrates cadmium [13,14,15,16]. This results in cadmium levels in placental tissue being four times higher than cadmium levels in maternal serum and six times higher than cadmium level in umbilical cord serum [17].

One potential mechanism by which prenatal cadmium exposure may impact early development is through the dysregulation of maternal and/or placental hormones that play essential roles in pregnancy physiology [18,19,20]. Dysregulation of hormone systems during pregnancy can result in, or be caused by, pregnancy complications such as pre-eclampsia and gestational diabetes [21,22]. Even in the absence of overt complications, because of the fetus’ reliance on maternal and placental substrates, endocrine disruption in pregnancy can impact both fetal and child growth and development [23]. To date, very few epidemiological studies have examined cadmium’s endocrine-disrupting potential during pregnancy. In the Puerto Rican PROTECT cohort (n = 815), in mid and late pregnancy, positive associations were observed between urinary cadmium and thyroid-stimulating hormone (TSH), but not other thyroid hormones or sex steroids [24]. Notably, in that study, maternal hormones were measured using enzyme immunoassay (ELISA) which lacks the sensitivity and specificity needed to evaluate hormones present in low concentrations in pregnancy, such as androgens [25,26].

The impacts on androgen production, in particular, are of interest as emerging data demonstrate that cadmium may dysregulate the hypothalamic–pituitary–gonadal (HPG) axis, resulting in altered androgen production rats [27]. Evidence from non-pregnant humans offers support for the cadmium-related disruption of androgen activity. In a study of healthy, reproductive-age U.S. women, a 0.1 µg/L increase in whole blood cadmium concentration was associated with 2.2% higher testosterone and 2.9% higher sex hormone-binding protein concentrations, as well as an 18% increased probability of a mild polycystic ovary syndrome (PCOS) phenotype characterized by high testosterone and anti-mullerian hormone concentrations [28]. Related work in that cohort additionally indicated associations between higher cadmium and higher follicular estradiol levels [29], as well as decreased mean follicle-stimulating hormone (FSH) levels [30]. Similar associations between cadmium exposure and HPG axis disruption have been reported in post-menopausal women [31,32] and men [33,34,35,36].

Cadmium’s endocrine-disrupting potential has also been demonstrated in experimental models, which have shown that cadmium exposure alters patterns of gonadotropin and androgen activity [37,38,39,40]. In a multigenerational study, gestational exposure of rats to cadmium (8 mg/kg) resulted in reduced serum testosterone in the male F1 offspring, but increased testosterone in the F2 males [41]. Likewise, in a study of pregnant mice, maternal cadmium exposure to cadmium-dosed drinking water (at 150 mg/L) throughout pregnancy inhibited placental progesterone synthesis and caused fetal growth restriction [42]. Furthermore, the authors reported that late gestational Cd exposure downregulated steroidogenic acute regulatory proteins (StAR) and 3ß-hydroxyl steroid dehydrogenase (3ß-HSD), critical enzymes for steroidogenesis. These findings suggest that cadmium may function as an environmental endocrine disruptor, but research on exposure during pregnancy remains quite limited. To advance our limited understanding of cadmium’s impact on the prenatal hormonal milieu, here we examined associations between urinary cadmium and serum sex steroid concentrations across pregnancy using data from a U.S. pregnancy cohort.

## 2. Materials and Methods

### 2.1. Study Overview and Population

The current analysis is based on data from participants in the Understanding Pregnancy Signals and Infant Development (UPSIDE) study based in Rochester, NY [43]. From 2015 to 2019, women with normal-risk pregnancies were recruited from outpatient obstetric clinics affiliated with the University of Rochester. Eligibility criteria included the following: <14 weeks gestation at enrollment, singleton pregnancy, age 18 or older, no history of psychotic illness or known substance use issues, and ability to communicate in English. Given the study’s focus on typical hormone activity in pregnancy, participants with major pre-existing endocrine disorders (e.g., polycystic ovary syndrome (PCOS), type II diabetes) were excluded from participation as were women with major medical conditions that put the pregnancy at high medical risk at the time of enrollment. A total of 326 pregnancies were recruited, of which 294 (90%) were retained through birth. Participants completed in-person study visits in each trimester which included biospecimen collection (urine) and questionnaire administration on topics including demographics, lifestyle factors, pregnancy, health history, and psychosocial measures. Study activities were approved by the University of Rochester and Rutgers University Institutional Review Boards (IRBs) and all participants signed informed consent prior to participation.

### 2.2. Exposure: Urinary Cadmium

Urine samples were collected during prenatal visits in each trimester (mean gestational age: 12.24 ± 1.27, 21.19 ± 1.77, and 31.31 ± 1.88 weeks) and for each sample, specific gravity was recorded as a measure of urine dilution. Samples were frozen at −80 °C pending cadmium analysis at the Environmental and Occupational Health Sciences Institute (EOHSI) at Rutgers University. At EOHSI, 250 µL of urine was aliquoted into metal-free polypropylene centrifuge tubes for cadmium analysis. The specimens were mixed with concentrated double-distilled nitric acid (100 µL), sonicated for 60 min, and digested (CEM microwave system, Matthews, NC) in 5 min intervals for 30 min at 300 W (75–100% power). The samples were diluted with Milli-Q water to produce a 5% nitric acid solution in preparation for quantification by inductively coupled plasma mass spectrometry (ICPMS) using a Nu Instruments Attom (Wrexham, UK) [44] (Wen et al., 2021). Standard ICPMS operation parameters were RF power of 1550 W, carrier gas flow of 1.00 L/min Ar, and nebulizer gas flow of ~32 psi Ar. Cadmium (Cd) in urine was quantified by measuring three replicate analyses at masses ^110^Cd, ^111^Cd, and ^112^Cd, and Mo-O polyatomic interferences on Cd were monitored by co-analyzing ^95^Mo and ^98^Mo. Daily calibration standards were prepared using metal concentrations (range 0.001–10 µg/L) with the instrument detection limit for cadmium being 0.005 µg/L. To account for instrument drift, we measured quality control standards (NIST Calibrant SM-1811-001) after every sixth sample (<5% RSD). The limit of quantification (LOQ) for cadmium was 0.04–0.08 µg/L. Adjustment for specific gravity was performed using the formula: [cadmium]_adj = [cadmium]_raw × [(SpG_median − 1)/(SpG − 1)], where SpG represents the specific gravity of the individual sample and SpG_median represents the median specific gravity of the cohort [45]. Samples with cadmium below the LOD (T1: n = 43, T2: n = 38, T3: n = 23) were assigned the value LOD/(square root of 2) per Hornung and Reed, 1989 [46].

### 2.3. Outcome: Maternal Sex Steroid Hormones

In each trimester, a 40 mL blood sample was collected and processed. Serum aliquots were frozen at –80 before being sent on dry ice to the Endocrine and Metabolic Research Laboratory at the Lundquist Institute at Harbor-UCLA Medical Center. Total testosterone (TT), free testosterone (fT), estrone (E1), estradiol [2], and estriol (E3) were measured via liquid chromatography with tandem mass spectrometry (LC–MS/MS) protocol, the gold standard, with adjustments to system parameters and runtime [47,48,49,50]. Analysis of TT was performed with a Shimadzu HPLC system (Columbia, MD, USA) and an Applied Biosystems API5500 LC–MS/MS (Foster City, CA, USA) including a Turbo Ion Spray source in positive ionization mode. Measurement of fT was done with equilibrium dialysis using labeled testosterone [51]. Estrogens were quantified using the Shimadzu HPLC system (Columbia, MD) with a triple quadrupole mass spectrometer serum from 1 participant who had values below detection limits for first trimester TT and fT; 17 participants had below detection levels or missing values for first trimester E3.

### 2.4. Covariates

Potential covariates were selected based on a directed acyclic graph (DAG) informed by the current literature [52,53,54] (Supplemental Figure S1). Data on covariates were collected through maternal questionnaires and medical record abstraction by trained study staff. Participants reported their age, race, and ethnicity at the time of recruitment. Maternal race and ethnicity were included in this analysis based on known disparities in cadmium exposure as well as the role of systemic racism in altering maternal perinatal physiology [52,55]. Given distributions, maternal race was categorized as White, Black, and other, while ethnicity was categorized as Hispanic or non-Hispanic. The highest level of education was determined based on prenatal questionnaires and classified as high school or less, some college or a bachelor’s degree, or postgraduate studies. Smoking was queried in each trimester and based on a limited number of smokers in the cohort, was dichotomized as any self-reported smoking during pregnancy versus none. Parity was classified as nulliparous versus multiparous based on maternal self-report. Maternal earliest first trimester pregnancy weight was used as a proxy for pre-pregnancy weight per convention in the field [56], and based on that, pre-pregnancy BMI was calculated as weight in kilograms divided by height in meters squared. We determined gestational age at each sample collection based on the American College and Obstetrician and Gynecologist protocols [57]. In general, crown-rump length at the first ultrasound was used for dating; when that was not available, the last menstrual period was used. Although this was a medically low-risk cohort at baseline, several participants developed pregnancy complications; major complications including preeclampsia, gestational diabetes, and preterm birth were abstracted from the medical record, and due to their low numbers, combined into an “any complications” variable. Biological sex of the infant was abstracted from the birth record.

### 2.5. Statistical Analysis

Descriptive statistics were calculated for the exposure, outcomes, and covariates of interest. Concentrations below the limit of detection for TT, fT, E3, and missing values for E3 were replaced by the LOD divided by the squared root of 2. Sex steroid hormones and cadmium concentrations were not normally distributed and were therefore log transformed. We examined correlations between cadmium and hormone concentrations using Spearman correlation. We also calculated a two-way mixed-effect intraclass correlation coefficient (ICC) to assess reliability in the urinary cadmium measurements [58]. For our primary analyses, to capitalize on the repeated measurements across pregnancy, we fit linear mixed models (LMMs) for each hormone with an unstructured correlation matrix, a fixed effect for cadmium, a smooth function for gestational age, and a random effect for each participant. We expressed results as the mean percent difference (∆%) in hormone concentrations associated with a ln-unit increased in cadmium. We evaluated potential confounders in multiple ways: *a priori* based on previous research, using DAGs, and assessing changes in effect estimates in bivariate models. Final covariates for TT and fT models included maternal age, gestational age at visit, maternal race, maternal ethnicity, highest maternal education, and pre-pregnancy BMI. Models for E1, E2, and E3 included those variables and were additionally adjusted for fetal sex, parity, maternal smoking during pregnancy. Secondarily, we fitted trimester-specific models, consisting of crude and adjusted linear regression models, examining the association between cadmium and each sex steroid hormone. In light of the substantial literature suggesting sex-specific impacts of cadmium on perinatal outcomes [59,60], we examined interaction terms between cadmium and fetal sex. Additionally, we stratified by fetal sex to better assess this potential effect modification [61]. Finally, we conducted two sensitivity analyses excluding the following (1) participants who reported any smoking in pregnancy; and (2) participants who had any major pregnancy complications (preeclampsia, gestational diabetes, preterm birth). Probability values (*p*-values) < 0.05 were considered statistically significant. Analyses were performed in SAS version 9.4.

## 3. Results

The 272 participants included in the current analysis were 29.14 ± 4.60 years old on average and 66% had a prior birth (Table 1). Few participants (7%) reported smoking during pregnancy and mean pre-pregnancy BMI was 28 ± 7 kg/m^2^. Most participants (64%) identified as White, with 24% identifying as Black, and 13% as a member of another race. Hispanic/Latina participants comprised 10% of the cohort. The highest level of educational attainment varied considerably with 36% of participants reporting having a high school diploma or less, 39% reporting some college or a bachelor’s degree, and 25% reporting postgraduate education. Of the infants born to the participating mothers in this study, 51% were male and 49% were female. In total, 94% of the pregnancies were full-term, and overall, mean gestational age was 39.5 weeks.

The percentage of samples with concentrations above the LOD for cadmium was 83.8%, 86.1%, and 91.5% in trimesters 1, 2, and 3, respectively (Table 2). Median specific gravity-adjusted cadmium concentrations were slightly higher in the first trimester (0.23 µg/L) than in trimesters 2 and 3 (0.15 and 0.17 µg/L, respectively). The correlation coefficients were T1 and T2: 0.58, T1 and T3: 0.42, T2 and T3: 0.54 (all < 0.05). The ICC for urinary cadmium concentrations was 0.44 (95%CI: 0.29, 0.56). Maternal TT concentrations were stable across gestation. Across trimesters 1, 2, and 3, median TT was 60, 63, and 62 ng/dL and median fT was 0.56, 0.48, and 0.50 ng/dL, respectively (Table 3). By contrast, all estrogen concentrations increased dramatically as pregnancy progressed. Across trimesters 1, 2, and 3, median E1 levels were 945, 3570, and 5855 pg/mL, median E2 was 1610, 5955, and 11,050 pg/mL, and median E3 was median 270, 3115, and 6615 pg/mL.

**Figure 1:**
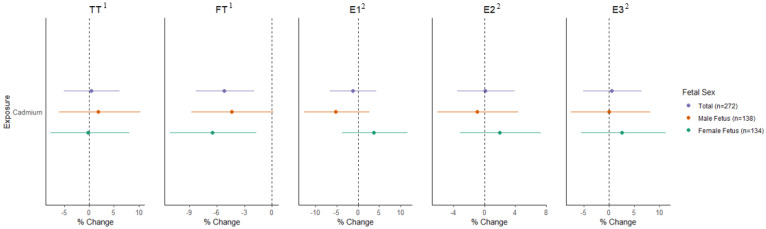
Linear mixed models examining associations between urinary cadmium and sex steroid hormone concentrations in pregnancy overall and stratified by fetal sex. ^1^ Adjusted for maternal age, gestational age deviation, maternal race, maternal ethnicity, highest maternal education, pre-pregnancy BMI. ^2^ Adjusted for maternal age, gestational age deviation, maternal race, maternal ethnicity, highest maternal education, pre-pregnancy BMI, fetal sex, parity, maternal smoking during.

In unadjusted LMMs examining urinary cadmium and sex steroid hormones across pregnancy, higher cadmium exposure was associated with significantly lower fT (%∆ = −4.61, 95%CI: −7.62, −1.51) as well as non-significantly lower E1 (%∆ = −2.86, 95%CI: −8.39, 3.02) and E2 (%∆ = −2.08, 95%CI: −6.22, 2.24) (Figure 1 and Supplemental Table S1). In adjusted models, the association between urinary cadmium and lower fT was strengthened (%∆ = −5.19, 95%CI: −8.33, −1.93), whereas the estimates for all other hormones were close to the null. In stratified analyses, inverse associations between cadmium and fT were observed in mothers carrying fetuses of both sexes, however results were only statistically significant in women carrying female fetuses (females: %∆ = −6.51, 95%CI: −11.11, −1.68; males: %∆ = −4.39, 95%CI: −8.75, 0.19; Figure 1). Interaction terms for cadmium*fetal sex were not significant for TT or fT (Supplemental Table S2). In contrast, interaction terms for cadmium*fetal sex were statistically significant for both E1 and E2 (*p* < 0.05). Associations between cadmium concentrations and lower fT were robust to the exclusion of smokers (n = 18; %∆ = −5.33, 95%CI: −8.57, −3.74) and participants with pregnancy complications (n = 35; %∆ = −3.74, 95%CI: −7.22, −0.13; Supplemental Table S3).

Secondarily, in linear regression models examining associations in individual trimesters, we observed inverse associations between cadmium and TT in early to mid-pregnancy (T1: %∆ = −8.30, 95%CI: −18.00, 2.54; T2: %∆ = 15.26, 95%CI: −25.15, −4.06) that were attenuated in late pregnancy (T3: %∆4.37, 95%CI: −9.94, 20.95; Supplemental Table S4). Patterns of association between cadmium and TT differed by fetal sex such that inverse associations were stronger in women carrying female fetuses in early to mid-pregnancy, after which associations were in the positive direction. By contrast, in males, associations were inverse (and non-significant) throughout pregnancy (Supplemental Table S4). Associations between cadmium and fT were strongest and only statistically significant in late pregnancy (T3: %∆ = −14.35, 95%CI: −19.75, −8.59), with associations observed in both sexes (females: %Δ = −17.70, 95%CI: −25.07, 9.61); males: %Δ = −11.23, 95%CI: −18.81, 2.96). No associations between cadmium and any estrogens were observed in trimester-specific models in the whole cohort or stratified by fetal sex (Supplemental Table S5).

## 4. Discussion

Overall, we observed that cadmium concentrations were associated with lower fT across gestation, with some indication of stronger associations in late pregnancy, as well as with lower TT in early–mid pregnancy. For the most part, results suggesting lower testosterone concentrations were consistent across participants carrying male and female fetuses and were robust to the exclusion of smokers and participants who developed pregnancy complications. In adjusted models, we observed no associations between cadmium concentrations and levels of any estrogen (estrone, estradiol, and estriol). Median urinary cadmium concentrations in our cohort were similar to those measured in other recent studies in pregnancies in the U.S. [62,63] as well as female NHANES participants [64].

In the only prior study on this topic based on the PROTECT cohort, no associations were observed between cadmium exposure and maternal sex steroid hormones, measured in mid- (16–20 weeks) and late (24–28 weeks) pregnancy [24]. Of note, in the prior study, TT was measured using ELISA which is considered a non-preferred method due to concerns regarding sensitivity and accuracy [65], whereas here we used highly sensitive instrumentation LC–MS/MS. Median TT levels were slightly higher in our cohort in mid-pregnancy (63.2 ng/dL vs. 51.5 ng/dL in PROTECT), but similar in late pregnancy (62.2 ng/dL vs. 60.7 ng/dL in PROTECT) [66]. Both studies observed mostly null associations between cadmium concentrations and TT (with the exception of mid-pregnancy associations in our study), despite differences in the hormone assays. An important innovation in the current work is that we additionally measured fT using LC–MS/MS with equilibrium dialysis [67]. Because fT represents only the small, biologically active fraction (1–4%) of TT, it is arguably most salient for studying impacts on fetal development such as impacts on growth [68], metabolism [69], and reproductive system development [70]. However, equilibrium dialysis is methodologically demanding and costly. As a result, many studies have relied solely upon TT measurements or have instead used formulas for calculating fT based on TT and binding protein concentrations (e.g., Vermeulen 1999 [71]). These calculations may or may not be valid in pregnancy due to changes in binding protein concentrations (most notably sex hormone binding globulin (SHBG)) as well as binding protein interference by rising estrogens [67].

Our finding that cadmium was associated with lower fT, even as TT concentrations remained largely unchanged, suggests the possibility of changes in binding protein concentrations following cadmium exposure. An estimated 78% of testosterone in women is bound by SHBG, a glycoprotein produced by the liver, with most of the remaining circulating testosterone bound by albumin (Yen and Jaffe textbook). The binding prevents diffusion into cells, thereby rendering the bound hormone fraction biologically inactive. The PROTECT study showed small positive associations between SHBG levels and cadmium concentrations in mid- and late pregnancy [24] but no other studies have examined this issue in pregnant populations. Additional studies in non-pregnant populations have examined this relationship, generally observing positive associations between cadmium concentrations and SHBG [28,33,34,35]. Some evidence additionally suggests that cations, including cadmium, can compete with sex steroids to bind to SHBG, which then feeds back to stimulate additional SHBG production [72]. In the absence of direct changes on testosterone production, an increase in SHBG associated with cadmium exposure would reduce fT availability, as suggested by our results.

Although the literature on cadmium and sex steroids in reproductive age women is sparse, in the BioCycle study of naturally cycling, reproductive age women in the U.S., higher baseline blood cadmium was associated with significantly higher TT and SHBG concentrations over two menstrual cycles of follow-up [28]. Higher baseline cadmium was additionally associated with non-significantly higher fT (calculated using a standard equation rather than equilibrium dialysis) and ultimately, increased odds of a mild PCOS-like phenotype [28]. Their findings of higher SHBG in relation to cadmium exposure are consistent with our findings of reduced fT. However, the higher TT and fT concentrations contrast with our results, which may be the result of differing dynamics of testosterone production and feedback in cycling versus pregnant individuals. Additional work in the BioCycle cohort suggested lower follicle-stimulating hormone (FSH), higher follicular estradiol, and increased menstrual cycle length in relation to higher cadmium exposure [29,30].

Associations between cadmium exposure and increased SHBG have also been observed in post-menopausal women and men. For example, in a study of post-menopausal Swedish women, Ali et al. (2014) reported positive associations between cadmium and SHBG, although they were non-significant after adjustment for covariates [31]. The study also reported differences in TT, estradiol, E2/TT ratio, and androstenedione, with some variation by cadmium matrix (blood or urine). Two additional studies of post-menopausal women examined associations between cadmium and sex steroids; however, they did not measure fT or SHBG. In a cross-sectional Japanese study, higher cadmium was associated with higher TT, but not E1 or DHEAS, and adjustment for covariates was limited [32]. In a Chinese study, blood cadmium was not associated with TT, E2, FSH, or LH in post-menopausal women; however in males, inverse associations between TT and SHBG were observed [35]. Finally, in two studies of male NHANES participants, cadmium was similarly associated with lower TT as well as lower SHBG, in contrast to the Chen et al. (2016) study [33,34,35].

Altered androgen and SHBG levels have been implicated in the development of some pregnancy complications, birth outcomes, and subsequent child health. For instance, in a large, nested case–control study, individuals who went on to develop gestational diabetes had higher early pregnancy testosterone and lower SHBG compared to controls [73]. Early pregnancy SHBG was inversely associated with fasting glucose levels later in mid-pregnancy; moreover, SHBG has been proposed as a clinical predictor of gestational diabetes mellitus [73,74]. Results of several studies have additionally suggested that higher maternal testosterone in pregnancy may be associated with low birthweight and preterm delivery [75,76]. Consistent with a Developmental Origins of Health and Disease (DOHaD) framework, prenatal hormones have also been linked to subsequent child outcomes including behavioral problems [47,77,78], autistic traits [79,80], and sex-stereotyped play behaviors [81]. However, research in this area, particularly among pregnant populations without endocrine conditions (e.g., PCOS) remains limited and additional work is needed to fully understand how prenatal hormones impact child health and development.

This epidemiological body of research suggests that cadmium may disrupt steroidogenic pathways as well as binding hormone production, though the patterns of association may vary depending on biological sex, life stage, and the hormonal milieu of the population of interest. Animal models provide some support to the human literature suggesting that prenatal cadmium exposure can alter the steroidogenic factor 1 (SF-1) and StAR in a multigenerational manner [41]. Experimental evidence also suggests that prenatal cadmium exposure impairs the cognitive function of the offspring by reducing placental derived E2 concentrations [82]. Additionally, the breast cancer literature suggests that cadmium can influence estrogen signaling by binding to estrogen receptors. In vitro, cadmium decreases the total number of available estrogen-binding sites in mammary tissue, binding to estrogen receptor GPR30 and ultimately promoting ER+ cancer cell growth [83]. As such, it has been suggested that cadmium can act as a metalloestrogen [83]. However, these mechanisms have received less attention outside of the context of cancer and in particular, in relation to pregnancy. Given the paucity of work on this topic in pregnancy, we posit that additional research is needed in light of evidence that disruption of sex steroid pathways in pregnancy may impact prenatal health, potentially with downstream impacts on child health.

Our study has several strengths worth noting. First, we measured urinary cadmium in each trimester allowing us to examine changes across pregnancy. Although excretion of cadmium into urine is considered a potential long-term marker of exposure, we observed some intra-individual variation in the concentrations across trimesters. We speculate that this may reflect a combination of varying exposure to cadmium, dynamic changes in maternal physiology (including increased renal blood flow and glomerular filtration rate), and/or continued growth of the placenta where cadmium can be sequestered leading to a new tissue compartment with impact on toxicokinetics across gestation. We also measured sex steroid hormones in each trimester, which was particularly important for the estrogens which are produced at much higher levels as pregnancy progresses. Participants with major endocrine disorders such as PCOS were excluded *a priori*, allowing us to examine associations between cadmium and sex steroids in women with no clinically diagnosed endocrine dysregulation at baseline. Finally, the use of LC–MS/MS with equilibrium dialysis to measure hormones, particularly testosterone, is an important advancement over the only prior study on this topic, which used ELISA [24]. Given the low concentrations of testosterone and other androgens in women, use of the more sensitive and specific LC–MS/MS assay is essential for accurate quantification.

At the same time, we note several limitations. First, this was a relatively healthy cohort at baseline which meant that we were underpowered to evaluate cadmium’s potential impacts on hormone levels through pregnancy complications such as preeclampsia; however, we note that our results were robust to the exclusion of the small fraction of participants with pregnancy complications. Second, we observed only moderate exposure to cadmium in this cohort and while concentrations were similar to those observed in other recent U.S. pregnancy cohorts [62,63], the relatively low levels may have hindered our ability to observe associations with altered endocrine profiles. Third, we only measured urinary cadmium in the current study; however, evaluating blood cadmium, which represents more recent exposures, may have provided additional insights into impacts on hormone dynamics across pregnancy. Additionally, we did not measure SHBG and albumin, which would provide more direct evidence of the mechanisms by which fT concentrations were decreased in relation to cadmium exposure in our study. Finally, maternal circulating hormones derive from multiple sources (mother, placenta, and fetus) and we cannot disentangle their relative contributions to overall hormone concentrations measured here.

In conclusion, in this relatively healthy pregnancy cohort with moderate cadmium exposure, higher cadmium concentrations were associated with lower fT concentrations across pregnancy, while few associations with estrogen concentrations were observed. These results show some consistencies with the literature on cadmium and sex steroid pathways in non-pregnant people including cycling women, post-menopausal women, and men; however, given the unique hormone environment of pregnancy, more research specific to pregnant people is needed. Additional work examining the disruption of gestational sex steroid hormone pathways by cadmium exposure is particularly salient given emerging evidence that prenatal sex steroids are increasingly recognized as important predictors of prenatal health and child development.

## Figures and Tables

**Table 1:** Characteristics of UPSIDE study participants (n = 272).

Variable	Mean ± SD	Min, Max
Maternal age (years)	29.14 ± 4.60	18, 41
Maternal pre-pregnancy BMI (kg/m^2^)	27.96 ± 7.17	15.31, 49.09
GA at trimester 1 visit (weeks)	12.24 ± 1.27	6.14, 14.43
GA at trimester 2 visit (weeks)	21.19 ± 1.77	18.14, 29.57
GA at trimester 3 visit (weeks)	31.31 ± 1.88	28.14, 39.00
GA at birth (weeks) ^1^	39.50 ± 1.43	33.00, 42.71
	**Frequency**	**Percentage**
Maternal race		
White	174	63.97
Black/African American	64	23.53
Other ^2^	34	12.50
Maternal ethnicity		
Non-Hispanic or non-Latino	246	90.44
Hispanic or Latino	26	9.56
Highest maternal Education		
High school or less	97	35.66
Some college, bachelors	106	38.97
Postgraduate	69	25.37
Parity		
Yes (>1)	181	66.54
No (0)	91	33.46
Fetal Sex		
Male	138	50.74
Female	134	49.26
Smoking during pregnancy		
No	254	93.38
Yes	18	6.62
Pregnancy complications		
Any	35	12.87
None	237	87.13
Term birth (>37 weeks)		
Yes	257	94.49
No	15	5.51

^1^ n = 270. ^2^ Other includes Asian, American Indian/Alaska Native, more than one raceBMI: body mass index, GA: gestational age, kg/m^2^: kilogram per meter squared.

**Table 2:** Maternal specific gravity-adjusted urinary cadmium concentrations (µg/L).

Cadmium	n	Percent > LOD ^1,2^	Min	Q1 (25%)	Median	Q3 (75%)	Max	Mean ± SD
Trimester 1	263	83.71%	0.04	0.16	0.23	0.36	2.82	0.30 ± 0.26
Trimester 2	267	86.14%	0.03	0.10	0.15	0.24	2.91	0.21 ± 0.26
Trimester 3	270	91.48%	0.03	0.12	0.17	0.24	3.75	0.22 ± 0.27

^1^ The LOD for Cd in urine was 0.08 µg/L. ^2^ Individual values below the LOD were imputed as LOD/(sqrt2).

**Table 3:** Descriptive statistics of maternal sex steroid hormones.

Hormone	Min	Q1 (25%)	Median	Q3 (75%)	Max	Mean ± SD
Trimester 1 (n = 270)						
TT (ng/dL)	8.80	37.10	59.90	88.30	303.00	70.54 ± 45.40
FT (ng/dL)	0.23	0.48	0.56	0.66	1.17	0.58 ± 0.16
E1 (pg/mL)	124.00	564.00	945.00	1430.00	5640.00	1156.21 ± 893.42
E2 (pg/mL)	356.00	1170.00	1610.00	2330.00	6390.00	1856.06 ± 995.65
E3 ^1^ (pg/mL)	10.00	116.00	270.50	486.00	1470.00	326.41 ± 265.56
Trimester 2 (n = 272)						
TT (ng/dL)	6.90	42.90	63.15	101.50	413.50	78.95 ± 54.68
FT (ng/dL)	0.19	0.43	0.48	0.56	0.93	0.50 ± 0.13
E1 (pg/mL)	423.00	2275.00	3570.00	5185.00	20,500.00	4215.79 ± 2986.52
E2 (pg/mL)	1750.00	4660.00	5955.00	7935.00	27,700.00	6509.85 ± 2968.77
E3 (pg/mL)	799.00	2340.00	3115.00	3890.00	11,900.00	3236.09 ± 1324.36
Trimester 3 (n = 270)						
TT (ng/dL)	9.60	36.90	62.15	101.00	349.50	77.72 ± 57.45
FT (ng/dL)	0.20	0.43	0.50	0.58	1.04	0.51 ± 0.15
E1 (pg/mL)	751.00	3640.00	5855.00	8130.00	37,700.00	6803.49 ± 5153.37
E2 (pg/mL)	2380.00	8980.00	11,050.00	14,200.00	31,900.00	11,966.78 ± 4872.46
E3 (pg/mL)	1030.00	5130.00	6615.00	8340.00	26,300.00	7167.04 ± 3368.12

TT: total testosterone, FT: free testosterone, E1: estrone, E2: estradiol, E3: estriol. ^1^ n = 255.

## Data Availability

No data are available. Once complete cohort data have been collect and cleaned, it will be made available to collaborators pending approved concept proposal, analysis plan and documentation of IRB approval.

## Supplementary Materials

The following supporting information can be downloaded at: https://www.mdpi.com/article/10.3390/toxics11070589/s1, Figure S1: Directed acyclic graph (DAG) for the association between prenatal cadmium exposure and maternal sex steroid hormones; Table S1: Linear mixed models examining associations between urinary cadmium and sex steroid hormone concentrations in pregnancy overall and stratified by fetal sex. Table S2: *p*-values for cross-product terms of cadmium by fetal sex in linear mixed models. Table S3: Adjusted linear mixed models examining associations between urinary cadmium and sex steroid hormone concentrations in pregnancy, excluding smokers and participants with pregnancy complications. Table S4: Adjusted^1^ linear regression models examining associations between maternal urinary cadmium and serum testosterone concentrations by trimester, overall and stratified by fetal sex. Table S5: Adjusted^1^ linear regression models examining associations between maternal urinary cadmium and estrogen concentrations by trimester.
