# Supplementary material for: Prenatal Cadmium Exposure and Maternal Sex Steroid Hormone Concentrations across Pregnancy
Source: Toxics. 2023 Jul 6;11(7):589. doi: 10.3390/toxics11070589 (PMC10384739; doi:10.3390/toxics11070589)
Supplement: Supplementary file 1 [file toxics-11-00589-s001.zip › toxics-2366668-supplementary.pdf]

toxics MDPI

Supplementary Materials: Prenatal Cadmium Exposure and Maternal Sex Steroid Hormone Concentrations across Pregnancy

Zorimar Rivera-Núñez, Megan Hansel, Camila Capurro, Danielle Kozlosky, Christina Wang, Cathleen L. Doherty, Brian Buckley, Pamela Ohman-Strickland, Richard K. Miller, Thomas G. O'Connor, Lauren M. Aleksunes and Emily S. Barrett

Table S1. Linear mixed models examining associations between urinary cadmium and sex steroid hormone concentrations in pregnancy overall and stratified by fetal sex.

		**Total Testosterone** (ng/dl)		**Free Testosterone** (ng/dl)		**Estrone** (pg/ml)		**Estradiol** (pg/ml)		**Estriol** (pg/ml)	
Cadmium (ppb)	n	Unadjusted Model %Δ (95% CI)	Adjusted Model[1] %Δ (95% CI)	Unadjusted Model %Δ (95% CI)	Adjusted Model[1] %Δ (95% CI)	Unadjusted Model %Δ (95% CI)	Adjusted Model[2] %Δ (95% CI)	Unadjusted Model %Δ (95% CI)	Adjusted Model[2] %Δ (95% CI)	Unadjusted Model %Δ (95% CI)	Adjusted Model[2] %Δ (95% CI)
All participants	272	0.02 (-5.37, 5.72)	0.38 (-5.10, 6.18)	**-4.61 (-7.62, -1.51)**	**-5.19 (-8.33, -1.93)**	-2.86 (-8.39, 3.02)	-1.24 (-6.57, 4.39)	-2.08 (-6.22, 2.24)	0.11 (-3.55, 3.90)	-0.85 (-6.52, 5.16)	0.50 (-5.09, 6.43)
Participants carrying male fetuses	138	2.38 (-5.42, 10.83)	1.83 (-6.02, 10.34)	-4.13 (-8.12, 0.03)	-4.39 (-8.75, 0.19)	-4.49 (-12.39, 4.13)	-5.28 (-12.69, 2.75)	-1.86 (-7.67, 4.32)	-0.99 (-6.10, 4.39)	0.82 (-6.70, 8.94)	-0.06 (-7.61, 8.11)
Participants carrying female fetuses	134	-2.82 (-10.09, 5.04)	-0.21 (-7.84, 8.05)	**-5.21 (-9.72, -0.48)**	**-6.51 (-11.11, -1.68)**	-0.16 (-7.65, 7.94)	3.65 (-3.70, 11.57)	-1.64 (-7.41, 4.50)	1.93 (-3.20, 7.33)	-2.77 (-10.93, 6.13)	2.49 (-5.55, 11.22)

[1] Adjusted for maternal age, gestational age deviation, maternal race, maternal ethnicity, highest maternal education, pre-pregnancy BMI.
[2] Adjusted for maternal age, gestational age deviation, maternal race, maternal ethnicity, highest maternal education, pre-pregnancy BMI, fetal sex, parity, maternal smoking during pregnancy.

Table S2. *P*-values for cross-product terms of cadmium by fetal sex in linear mixed models.

**Outcome**	**Exposure**	**Fetal Sex (*p*-value)**
Total Testosterone[1]	Cadmium	0.76
Free Testosterone[1]	Cadmium	0.98
Estrone[2]	Cadmium	0.04
Estradiol[2]	Cadmium	0.04
Estriol[2]	Cadmium	0.35

[1] Adjusted for maternal age, gestational age deviation, maternal race, maternal ethnicity, highest maternal education, pre-pregnancy BMI.

[2]Adjusted for maternal age, gestational age deviation, maternal race, maternal ethnicity, highest maternal education, pre-pregnancy BMI, fetal sex, parity, maternal smoking during pregnancy.

Table S3. Adjusted linear mixed models examining associations between urinary cadmium and sex steroid hormone concentrations in pregnancy, excluding smokers and participants with pregnancy complications.

	**Total Testosterone[1]**	**Free Testosterone[1]**	**Estrone[2]**	**Estradiol[2]**	**Estriol[2]**
Cadmium (ppb)	%Δ (95% CI)	%Δ (95% CI)	%Δ (95% CI)	%Δ (95% CI)	%Δ (95% CI)
Excluding smokers[3] (n=254)	1.38 (-4.20, 7.29)	**-5.33 (-8.57, -1.98)**	-0.39 (-5.93, 5.47)	0.07 (-3.70, 3.99)	0.55 (-5.14, 6.59)
Excluding participants with pregnancy complications (n=237)	-2.26 (-8.00, 3.85)	**-3.74 (-7.22, -0.13)**	-1.32 (-6.86, 4.56)	-0.13 (-4.03, 3.92)	1.17 (-4.91, 7.65)

[1]Adjusted for maternal age, gestational age deviation, maternal race, maternal ethnicity, highest maternal education, pre-pregnancy BMI.
[2]Adjusted for maternal age, gestational age deviation, maternal race, maternal ethnicity, highest maternal education, pre-pregnancy BMI, fetal sex, parity, maternal smoking during pregnancy.
[3]Not adjusted for maternal smoking doing pregnancy.

Table S4. Adjusted[1] linear regression models examining associations between maternal urinary cadmium and serum testosterone concentrations by trimester, overall and stratified by fetal sex.

Cadmium (ppb)	**Total Testosterone**			**Free Testosterone**		
Overall	Trimester 1 (n=261)	Trimester 2 (n=264)	Trimester 3 (n=266)	Trimester 1 (n=261)	Trimester 2 (n=264)	Trimester 3 (n=266)
%Δ (95% CI)	-8.30 (-18.00, 2.54)	**-15.26 (-25.15, -4.06)**	4.37 (-9.94, 20.95)	-3.75 (-8.64, 1.40)	0.96 (-4.27, 6.48)	**-14.35 (-19.75, -8.59)**
Participants carrying male fetuses	Trimester 1 (n=133)	Trimester 2 (n=134)	Trimester 3 (n=136)	Trimester 1 (n=133)	Trimester 2 (n=134)	Trimester 3 (n=136)
%Δ (95% CI)	-2.20 (-16.31, 14.29)	-13.73 (-28.40, 3.95)	-8.68 (-26.51, 13.46)	-3.69 (-10.40, 3.51)	3.48 (-3.73, 11.24)	**-11.23 (-18.81, -2.96)**
Participants carrying female fetuses	Trimester 1 (n=128)	Trimester 2 (n=130)	Trimester 3 (n=130)	Trimester 1 (n=128)	Trimester 2 (n=130)	Trimester 3 (n=130)
%Δ (95% CI)	-13.85 (-27.21, 1.95)	**-15.98 (-29.31, -0.14)**	19.29 (-3.01, 46.72)	-4.71 (-12.19, 3.41)	-1.23 (-9.04, 7.25)	**-17.70 (-25.07, -9.61)**

[1]Adjusted for maternal age, gestational age at trimester visit, maternal race, maternal ethnicity, highest maternal education, pre-pregnancy BMI.

Table S5. Adjusted[1] linear regression models examining associations between maternal urinary cadmium and estrogen concentrations by trimester.

**Cadmium (ppb)**	**Estrone**			**Estradiol**			**Estriol**		
Overall	Trimester 1 (n=261)	Trimester 2 (n=264)	Trimester 3 (n=266)	Trimester 1 (n=261)	Trimester 2 (n=264)	Trimester 3 (n=266)	Trimester 1 (n=239)	Trimester 2 (n=264)	Trimester 3 (n=266)
%Δ (95% CI)	-1.71 (-13.67, 11.92)	-3.80 (-15.60, 9.66)	-4.69 (-18.27, 11.15)	2.78 (-5.91, 12.28)	-4.12 (-11.16, 3.48)	-2.00 (-10.01, 6.72)	-1.31 (-18.49, 19.49)	0.45 (-6.92, 8.40)	3.31 (-5.69, 13.16)
Participants carrying male fetuses	Trimester 1 (n=133)	Trimester 2 (n=134)	Trimester 3 (n=136)	Trimester 1 (n=133)	Trimester 2 (n=134)	Trimester 3 (n=136)	Trimester 1 (n=122)	Trimester 2 (n=134)	Trimester 3 (n=136)
%Δ (95% CI)	-2.64 (-17.53, 14.95)	-9.07 (-25.01, 10.24)	-4.12 (-23.85, 20.74)	-0.98 (-12.01, 11.43)	-5.58 (-15.64, 5.68)	-6.05 (-17.68, 7.22)	-6.85 (-29.66, 23.36)	-0.67 (-9.89, 9.49)	3.15 (-10.56, 18.96)
Participants carrying female fetuses	Trimester 1 (n=128)	Trimester 2 (n=130)	Trimester 3 (n=130)	Trimester 1 (n=128)	Trimester 2 (n=130)	Trimester 3 (n=130)	Trimester 1 (n=1117)	Trimester 2 (n=130)	Trimester 3 (n=130)
%Δ (95% CI)	-1.63 (-20.67, 21.98)	3.66 (-13.83, 24.69)	-4.36 (-23.15, 19.03)	8.59 (-5.74, 25.10)	-1.42 (-11.49, 9.80)	1.49 (-9.41, 13.71)	1.10 (-24.01, 34.50)	1.50 (-10.33, 14.90)	3.66 (-8.40, 17.32)

[1]Adjusted for maternal age, gestational age at trimester visit, maternal race, maternal ethnicity, highest maternal education, pre-pregnancy BMI, fetal sex, parity, maternal smoking during pregnancy.

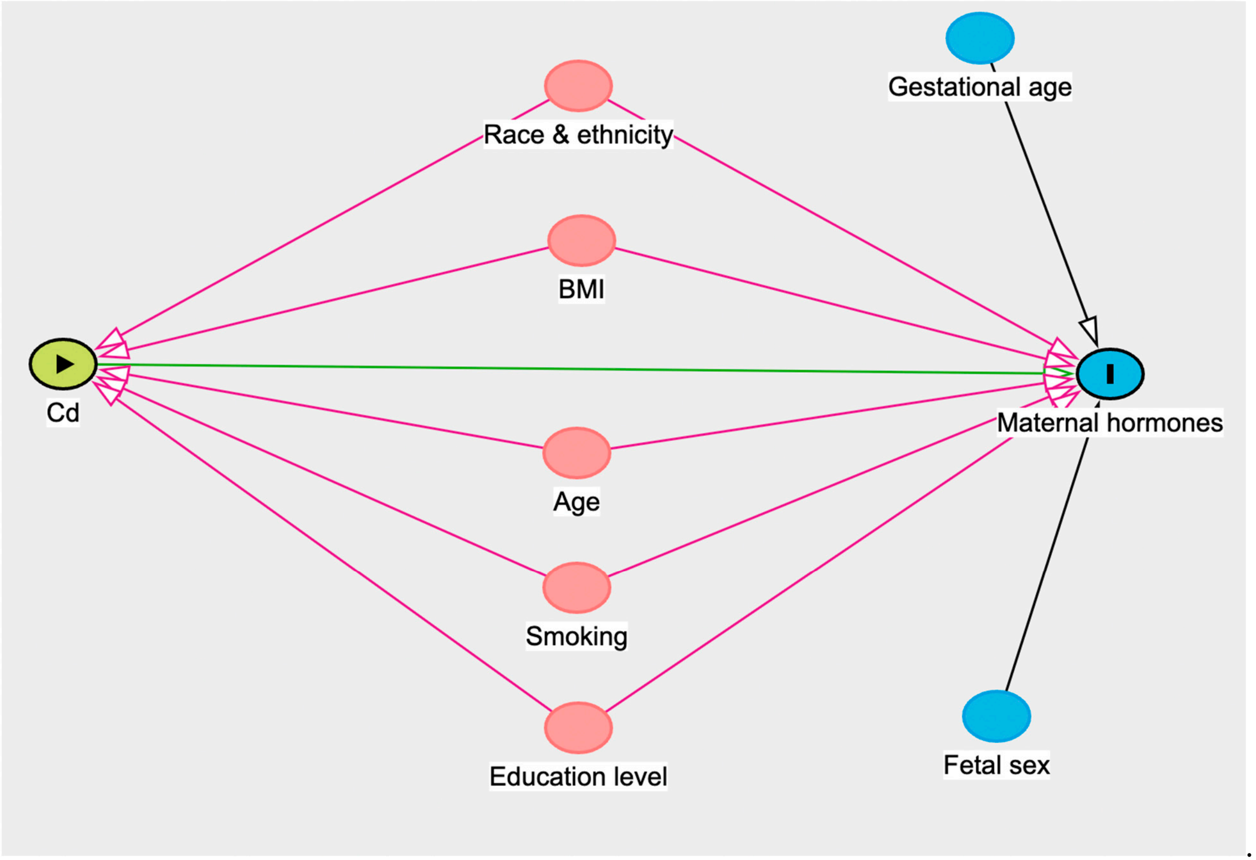

Figure S1. Directed acyclic graph (DAG) for the association between prenatal cadmium exposure and maternal sex steroid hormones.
